# A Retrospective Study on the Incidence of Pulmonary Embolism in Immobilized Spinal Cord Injury Patients

**DOI:** 10.7759/cureus.47691

**Published:** 2023-10-25

**Authors:** Kean Loong Ong, Amir Fariz Zakaria, Nur Azlin Zainal Abidin

**Affiliations:** 1 Spine Unit, Department of Orthopedics, Hospital Sungai Buloh, Ministry of Health Malaysia, Kuala Lumpur, MYS

**Keywords:** prolonged immobilization, deep vein thrombosis (dvt), thromboprophylaxis, pulmonary embolism, spinal cord injury

## Abstract

Introduction

The development of pulmonary embolism (PE) remains the main cause of morbidity and mortality in patients with spinal cord injuries. Multiple factors have been identified to predict the presence of PE in spinal cord injury patients, however, local data is scarce. The objective of this study is to evaluate the incidence of PE among spinal cord injury patients and analysis of predictive risk factors.

Material and methods

This is a retrospective study among spinal cord injury patients admitted to a tertiary hospital in Malaysia between January 2018 and December 2019. All spinal cord injury patients with symptoms suggestive of venous thromboembolism (VTE) such as tachycardia and shortness of breath were included in this study. Demographic data such as age, gender, types of VTE prophylaxis (mechanical or chemical), and radiological findings of computerized tomography pulmonary angiogram (CTPA) were analyzed.

Results

A total of 373 patients were included in the study. 301 of them have undergone spinal surgery. There were 251 males (75.75%) and 124 females (33.24%). The mean age of the subjects was 48.63 ± 17.45 years. The mean length of hospital stay is 42.74 ± 41.51 days. 151 (40.48%) patients received DVT prophylaxis while 222 (59.52%) patients did not. The incidence of PE among spinal cord injury patients with VTE symptoms is 25 patients (6.70%). Of that, 15 patients received medical treatment only, seven received mechanical and pharmacological prophylaxis and three did not receive any prophylaxis. In the variable analyses, none of the variables (age and types of prophylaxis) could significantly predict the occurrence of VTE.

Conclusion

PE is a frequent complication in immobilized spinal cord injury patients despite receiving thromboprophylaxis treatment. No strong predictors for PE were identified in the present study. However, patients who received any type of thromboprophylaxis was found to be statistically significant when compared to patients who did not receive any thromboprophylaxis in term of the presence of PE.

## Introduction

Venous thromboembolism (VTE) involves the formation of a thrombus in a vein that may dislodge from its site of origin to travel in the blood, a phenomenon called embolism. The development of pulmonary embolism (PE) remains the main cause of morbidity and mortality in patients with spinal cord injuries. The prevalence of deep vein thrombosis and PE in spinal cord injury was estimated as 14%-100% and 9%-90%, respectively [1]. The incidence of VTE remained low (1.8% for DVT, 2.4% for PE, and 4.2% overall) as reported by Chowdhury et al. in polytrauma patients [2].

The higher risk of spinal cord injury is due to the presence of all three components of the Virchow triads. Over a century ago, Rudolf Virchow described three factors that are critically important in the development of venous thrombosis: (1) venous stasis, (2) activation of blood coagulation, and (3) vein damage. These factors have come to be known as the Virchow triad [1]. Clinical diagnosis of VTE is usually unreliable and an investigative module requirement is almost necessary to successfully diagnose the condition [1].

Studies reported that computed tomography imaging is an effective diagnostic tool with up to 83% sensitivity and 96% specificity [3,4]. The prevention of VTE is important as PE is one of the leading causes of death in spinal cord injury patients [5].

Even though the incidence of VTE in patients with spinal cord was found to be decreasing from the 1970s to the 1990s, there is data paucity regarding the incidence of the conditions and no reports were documented [6]. A few predictors have been proposed, but they are not sufficient and lack strong evidence to support the findings. Despite some treatments and prophylaxis being proposed by physicians, further studies are required to evaluate the true effectiveness of recommended physical prophylaxis [7]. The objective of this study was to evaluate the incidence of VTE in patients with spinal cord injury and its predicting factors.

## Materials and methods

This is a retrospective study designed among orthopedic patients with spinal cord injury diagnosed with VTE in Hospital Sungai Buloh from January 1, 2018 to December 31, 2019. The objectives of this study were to determine the incidence of VTE among patients with spinal cord injuries, as well as VTE’s association with patients’ characteristics such as age, gender, weight, and height and type of VTE prophylaxis (mechanical such as TED stocking or pneumatic calf pump or both and any form of chemical prophylaxis such as low molecular weight heparin or unfractionated heparin). All patients undergo a computerized tomography angiogram (CTA) once patients present with signs and symptoms the patient presents either in the ward or at initial presentation to the Emergency Department.

The inclusion criteria entailed all patients with spinal cord injuries aged above 10 years old diagnosed with VTE during the study period regardless of the level of injury. The exclusion criteria are patients with VTE diagnosed with spinal cord injury secondary to other conditions such as tumor or metastasis, patients younger than 10 years of age, and patients with hematological diseases and bleeding disorders. Ethical approval for the study was obtained from the Medical Research and Ethics Committee of the institution.

Data analysis was conducted using the Statistical Package for Social Science (SPSS Version 25 (IBM Corp., Armonk, NY)). The normality of the data will be assessed using appropriate statistical tests to ensure that the underlying assumptions of normal distribution are met. Categorical data were presented as frequency (n) and percentage (%) while continuous data were summarized as mean ± standard deviation (SD) and range. Chi-square test and independent t-test were conducted to determine the association between the patient’s characteristics, other confounding factors, and the presence of VTE.

## Results

A statistical normality test was done, and all data was found to be normally distributed. Patients’ demographic data are presented in Table 1. A total of 373 patients with spinal cord injuries were included in this study. 251 (67.3%) being male and 122 (32.7%) being female. The average age is 48.63 years old. Only 151 patients have received VTE prophylaxis.

**Table 1:** Patient’s characteristics (n = 373)

Variables	n (%)
Gender	
Male	251 (75.75)
Female	124 (33.24)
Age (years), mean±SD	48.63 ± 17.45
DVT Prophylaxis	
Yes	151 (40.48)
No	222 (59.52)

Table 2 detailed the patients who were diagnosed with or without PE and their demographic data with the type of VTE prophylaxis used. Age was found to be not a confounding factor with p-value more than 0.05.

**Table 2:** Association between patient’s characteristics and PE (n = 373)

Variables	PE		P-value
	Present (n = 25)	Absent (n = 348)	
Gender			
Male	20 (80%)	231 (66.4%)	0.151
Female	5 (20%)	117 (33.6%)	
Age (years)	50.5 ± 19.6	48.4 ± 17.2	0.316
Weight (kg)	73.3 ± 20.8	78.0 ± 21.9	0.672
Height (cm)	161.3 ± 6.7	161.9 ± 7.7	0.865
Length of Hospital Stay (days)	55.8 ± 55.1	36.2 ± 26.3	0.205
VTE Prophylaxis			
Medicine	15 (4.0%)	64 (17.2%)	0.095
Physical	0 (0%)	20 (5.4%)	
Medicine+ Physical	7 (1.8%)	45 (12.1%)	
No Prophylaxis	22 (5.9%)	200 (53.6%)	0.001
Presence of Surgical Intervention			
Operation	18 (4.8%)	283 (75.9%)	0.380
Non-operative	7 (1.8%)	65 (17.5%)	

**Figure 1:**
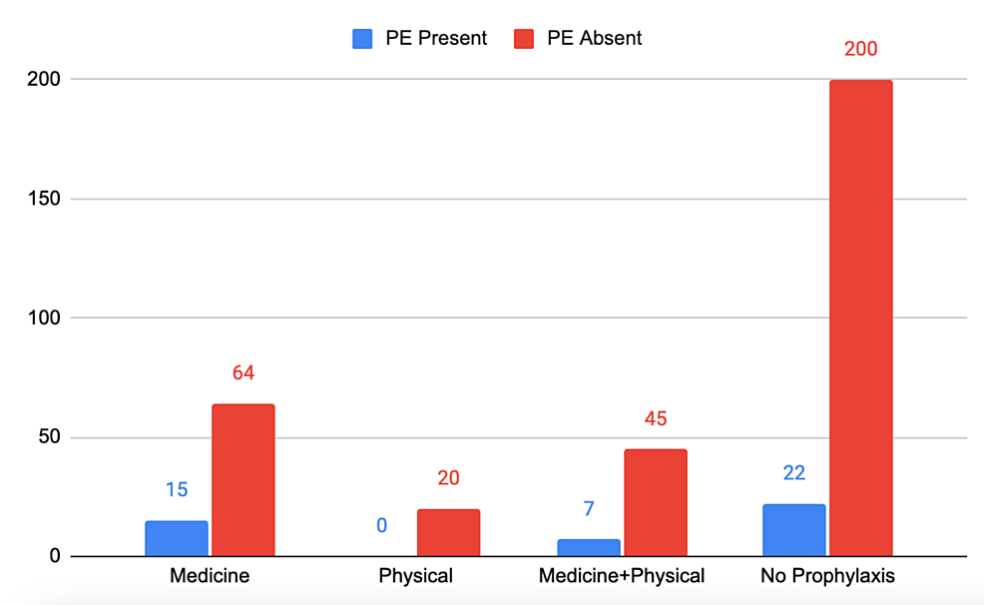
Number of patients with type of VTE prophylaxis and the presence of PE

Furthermore, cross-tabulation of the Chi-square test with the relationship between the type of VTE prophylaxis and PE has yielded a p-value of 0.095. However, a significant difference was found with the use of VTE prophylaxis in avoiding VTE. Surgical intervention did not have a correlation with the presence of VTE (p = 0.380) (Figure 1)*.*

## Discussion

In this study, there were more male preponderance as compared to females. The method used in diagnosing VTE was by identifying patients who developed PE based on the CT pulmonary angiogram. Based on the analyzed data, most of the patients were given a combination of mechanical and chemical DVT prophylaxis. The prevalence of VTE among the 151 patients given DVT prophylaxis was 14.5% while other 85.5% did not develop VTE. Rathore et al. found a prevalence rate of 12.8% for VTE in spinal cord injury patients who were not given DVT prophylaxis [8]. Siavash et al. documented that 11% of spinal cord injury subjects who were on prophylaxis developed symptomatic VTE, but none had fatal PE. Additionally, Siavash et al. found no statistical difference in the prevalence rate of VTE between the group (13%) that received low molecular weight heparin and those that received increased intensity low molecular weight heparin (8.3%) [9]. Aito et al. studied the difference in VTE incidence in patients who received thromboprophylaxis within the first 72 hours of trauma compared to those who were admitted between eight and 28 days. They found that no PE was reported in the early admission group while 26% of the late group developed DVT [10]. Myllynen et al. studied the incidence of DVT in paralyzed versus non-paralyzed but immobilized spinal cord injury patients and found that all paralyzed patients were diagnosed with DVT by using a 125l-labeled fibrinogen test confirmed with venography [11].

The present study compares the incidence rate between those receiving different forms of prophylaxis. None of the potential predictors for VTE in this study (gender, age, length of hospital stays, weight/height) was associated with the prevalence of VTE in spinal cord injury patients in agreement with some of the findings found in other studies [5]. Jones et al. in their study have found male preponderance, complete paralysis, African American, and more than three comorbidities to be the confounding factors to predict the presence of VTE after spinal cord injury [12]. We have found that in this study, the use of VTE prophylaxis is found to be statistically significant (p < 0.001) when associated with the presence of VTE by CTA. This could mean that the signs and symptoms of patients who prompted suspicion of VTE and the initiation of VTE prophylaxis are able to detect real VTE events. Surgery was not found to be a confounding factor when associated with the presence of VTE.

Different generic use of low molecular weight heparin was initially considered to be a factor. However, the factor did not correlate with the rate of VTE in the current study as there was no preponderance of developing VTE with different generic drug groups of low molecular weight heparin.

There are several limitations to this study. First, this is a retrospective study, and the results may be subject to bias, incomplete information, or misdiagnosis. Second, confounding co-interventions such as the use of intermittent pneumatic compression could not be evaluated as it was not recorded for all patients. Hence, it was assumed that all the patients were treated according to the CPG guideline protocol. Third, the present data and results were obtained from a single institution center, other neighboring medical centers have few reported incidences of VTE; however, these findings could not be compared to the results from the present study center. Fourth, our sample data may be small to identify the predictive risk factors for VTE, and a larger sample size involving another center with a longer duration of research period may be needed.

## Conclusions

PE is a serious complication in immobilized orthopedics patients due to spinal cord injuries despite receiving thromboprophylaxis treatment. Age, weight, height, length of hospital stays, types of DVT prophylaxis, and whether patients have undergone surgical intervention were not identified as strong predictors for PE in this study. However, patients who received thromboprophylaxis in chemical, physical or both have been found to be statistically significant when compared to patients who did not receive any thromboprophylaxis. A larger prospective study is needed to further investigate the predictors for VTE in patients with spinal cord injury.

## References

[1] Turpie AG, Chin BS, Lip GY (2002). Venous thromboembolism: pathophysiology, clinical features, and prevention. BMJ.

[2] Chowdhury S, Alrawaji F, Leenen LP (2021). Incidence and nature of lower-limb deep vein thrombosis in patients with polytrauma on thromboprophylaxis: a prospective cohort study. Vasc Health Risk Manag.

[3] Doğan H, de Roos A, Geleijins J, Huisman MV, Kroft LJ (2015). The role of computed tomography in the diagnosis of acute and chronic pulmonary embolism. Diagn Interv Radiol.

[4] Rahaghi FN, Minhas JK, Heresi GA (2018). Diagnosis of deep venous thrombosis and pulmonary embolism: new imaging tools and modalities. Clin Chest Med.

[5] Waring WP, Karunas RS (1991). Acute spinal cord injuries and the incidence of clinically occurring thromboembolic disease. Paraplegia.

[6] Heit JA, Kobbervig CE, James AH, Petterson TM, Bailey KR, Melton LJ 3rd (2005). Trends in the incidence of venous thromboembolism during pregnancy or postpartum: a 30-year population-based study. Ann Intern Med.

[7] Teasell RW, Hsieh JT, Aubut JA, Eng JJ, Krassioukov A, Tu L (2009). Venous thromboembolism after spinal cord injury. Arch Phys Med Rehabil.

[8] Rathore MF, Hanif S, New PW, Butt AW, Aasi MH, Khan SU (2008). The prevalence of deep vein thrombosis in a cohort of patients with spinal cord injury following the Pakistan earthquake of October 2005. Spinal Cord.

[9] Piran S, Schulman S (2016). Incidence and risk factors for venous thromboembolism in patients with acute spinal cord injury: a retrospective study. Thromb Res.

[10] Aito S, Pieri A, D'Andrea M, Marcelli F, Cominelli E (2002). Primary prevention of deep venous thrombosis and pulmonary embolism in acute spinal cord injured patients. Spinal Cord.

[11] Myllynen P, Kammonen M, Rokkanen P, Böstman O, Lalla M, Laasonen E (1985). Deep venous thrombosis and pulmonary embolism in patients with acute spinal cord injury: a comparison with nonparalyzed patients immobilized due to spinal fractures. J Trauma.

[12] Jones T, Ugalde V, Franks P, Zhou H, White RH (2005). Venous thromboembolism after spinal cord injury: incidence, time course, and associated risk factors in 16,240 adults and children. Arch Phys Med Rehabil.

